# ReQTL: identifying correlations between expressed SNVs and gene expression using RNA-sequencing data

**DOI:** 10.1093/bioinformatics/btz750

**Published:** 2019-10-07

**Authors:** Liam F Spurr, Nawaf Alomran, Pavlos Bousounis, Dacian Reece-Stremtan, N M Prashant, Hongyu Liu, Piotr Słowiński, Muzi Li, Qianqian Zhang, Justin Sein, Gabriel Asher, Keith A Crandall, Krasimira Tsaneva-Atanasova, Anelia Horvath

**Affiliations:** 1 Department of Medical Oncology, Dana-Farber Cancer Institute, Boston, MA 02215, USA; 2 Cancer Program, The Broad Institute of MIT and Harvard, Cambridge, MA 02142, USA; 3 Biochemistry and Molecular Medicine, McCormick Genomics and Proteomics Center; 4 Computer Applications Support Services, School of Medicine and Health Sciences, The George Washington University, Washington, DC 20037, USA; 5 Department of Mathematics & Living Systems Institute, University of Exeter, Exeter EX4 4QD, UK; 6 Department of Biochemistry and Molecular Medicine; 7 Department of Biostatistics and Bioinformatics, School of Medicine and Health Sciences, George Washington University, Washington, DC 20037, USA; 8 Computational Biology Institute, Department of Biostatistics and Bioinformatics, Milken Institute School of Public Health, The George Washington University, Washington, DC 20052, USA; 9 EPSRC Centre for Predictive Modelling in Healthcare, University of Exeter, Exeter EX4 4QJ, UK; 10 Department of Pharmacology and Physiology, School of Medicine and Health Sciences, The George Washington University, Washington, DC 20037, USA

## Abstract

**Motivation:**

By testing for associations between DNA genotypes and gene expression levels, expression quantitative trait locus (eQTL) analyses have been instrumental in understanding how thousands of single nucleotide variants (SNVs) may affect gene expression. As compared to DNA genotypes, RNA genetic variation represents a phenotypic trait that reflects the actual allele content of the studied system. RNA genetic variation at expressed SNV loci can be estimated using the proportion of alleles bearing the variant nucleotide (variant allele fraction, VAF_RNA_). VAF_RNA_ is a continuous measure which allows for precise allele quantitation in loci where the RNA alleles do not scale with the genotype count. We describe a method to correlate VAF_RNA_ with gene expression and assess its ability to identify genetically regulated expression solely from RNA-sequencing (RNA-seq) datasets.

**Results:**

We introduce ReQTL, an eQTL modification which substitutes the DNA allele count for the variant allele fraction at expressed SNV loci in the transcriptome (VAF_RNA_). We exemplify the method on sets of RNA-seq data from human tissues obtained though the Genotype-Tissue Expression (GTEx) project and demonstrate that ReQTL analyses are computationally feasible and can identify a subset of expressed eQTL loci.

**Availability and implementation:**

A toolkit to perform ReQTL analyses is available at https://github.com/HorvathLab/ReQTL.

**Supplementary information:**

Supplementary data are available at *Bioinformatics* online.

## 1 Introduction

Quantitative trait loci (QTL)-based approaches have served as a major tool to uncover genetic variants regulating phenotypic features. QTL methods have been successfully applied to a variety of molecular traits, including gene expression (eQTL), splicing (sQTL), protein expression (pQTL), methylation (meQTL), chromatin accessibility (chQTL/caQTL) and histone modification (hQTL/cQTL) (Aguet *et al.*, 2017; De Almeida *et al.*, 2018; Albert and Kruglyak, 2015; Atak *et al.*, 2013; Brandt and Lappalainen, 2017; Heinig, 2018; Ko *et al.*, 2017; Li *et al.*, 2015; Odhams *et al.*, 2017; Weiser *et al.*, 2014; Winter *et al.*, 2018). To correlate genetic variants with a trait of interest, the vast majority of these methods utilize the genotypes obtained through DNA analysis for each single nucleotide variant (SNV) locus.

With the recent advances in methods to call SNVs from RNA-seq data (Van der Auwera, *et al.*, 2013; Deelen *et al.*, 2015; Horvath, *et al.*, 2013; Piskol *et al.*, 2013), eQTL studies using genotypes inferred from RNA-seq have emerged. These studies have demonstrated sufficient power to identify genetically regulated expression and have generated valuable sets of genetic data (Tung *et al.*, 2015). Importantly, such approaches enable QTL analyses using only RNA-seq data, making it possible to explore datasets for which matched DNA data is not available.

For diploid genomes, a commonly used measure for quantitation of variant alleles at expressed SNV loci in RNA is the variant allele fraction (VAF_RNA_). VAF_RNA_ can be estimated from RNA-seq data (VAF_RNA_ = **n**_var_/(**n**_var_ + **n**_ref_)), where **n**_var_ and **n**_ref_, are the variant and reference sequencing read counts, respectively (Movassagh *et al.*, 2016). In contrast to the categorical genotypes (DNA-variant allele count of 0, 1 and 2, corresponding to homozygous-reference, heterozygous and homozygous-variant genotype, respectively), VAF_RNA_ is a continuous measure which allows for precise allele quantitation in loci where the RNA alleles do not scale with the genotype count. These include SNV loci under allele specific expression (ASE-SNVs, which are often subject to expression regulation, or are co-allelic with expression regulatory SNVs) and loci subjected to RNA-editing. Both ASE and RNA-editing can be extensively regulated through RNA-binding molecules, including those involved in transcript generation, processing, stability and structural maintenance (Casamassimi *et al.*, 2017; Chess, 2016; Do *et al.*, 2017; Eisenberg and Levanon, 2018; Gagnidze *et al.*, 2018; Imprialou *et al.*, 2017; Moreno-Moral *et al.*, 2017; Vandiedonck, 2018). Assessment of correlations between VAF_RNA_ and gene expression can be potentially used to assess the above regulatory relationships.

Herein, we propose a method to assess SNV-gene expression relationships based on VAF_RNA_-derived information on genetic variation; we call the method ReQTL (**R**NA-**eQTL**). We have based our model on the same assumption underlying eQTLs: if a given variant affects the expression of a given gene, the expression of this gene scales with the number of alleles harboring the variant of interest. This assumption intuitively encompasses both DNA-mediated effects, where the RNA allele abundance scales with the DNA-allele count, and effects resulting from solely RNA-mediated interactions. We note that ReQTL analyses are confined to expressed SNVs and do not identify transcriptionally silent regulatory loci. As a result, ReQTL analyses are expected to capture only a subset of the eQTL loci, and are likely to highlight SNVs that are co-allelic (in phase) with an actual regulatory or causative variant.

**Fig. 1.**
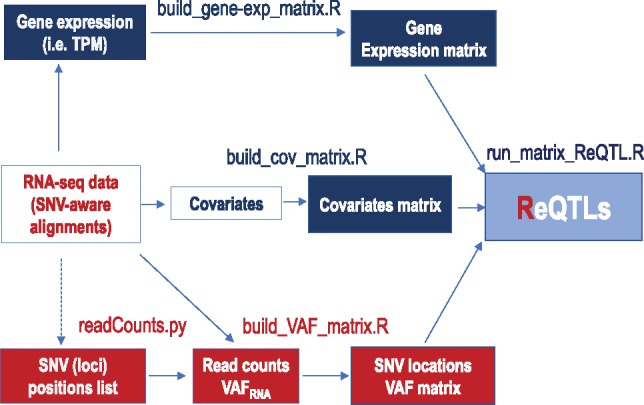
Major steps of the ReQTL analyses (differences from eQTL analysis are outlined in red). SNV-aware alignments are used to generate gene expression data; TPM values are quantile transformed and used to generate the gene expression matrix (exemplified by build_gene-exp_matrix.R). Lists of genomic positions can be built using any custom set of positions of interest (i.e. dbSNP). Alternatively, lists of genomic positions can be generated through variant call and subsequent retainment of the unique variant genomic loci across the sample set. At each genomic position in the list, the reference and variant number of RNA-seq reads are counted from the alignments and used to estimate VAF_RNA_ in each individual sample from the set (https://github.com/HorvathLab/NGS/tree/master/readCounts). The VAF_RNA_ estimations are used to build the VAF matrix (exemplified by build_VAF_matrix.R). Covariates can be accounted for by using approaches similar to the ones used in eQTL analyses. The three matrices are then used as input for Matrix eQTL (exemplified by run_matrix_ReQTL.R). (Color version of this figure is available at *Bioinformatics* online.)

ReQTL analyses can be run directly on computational platforms designed for eQTL analysis. We exemplify an implementation of ReQTL using the popular software Matrix eQTL (Shabalin, 2012) on RNA-seq data obtained from the Genotype-Tissue Expression (GTEx) project (www.gtexportal.org, phs000424.v7), from three different tissue types: Nerve-Tibial, Skin-Sun-Exposed (lower leg) and Skin-Not-Sun-Exposed (suprapubic). The proposed pipeline (Fig. 1) employs publicly available packages for processing of sequencing data, and a toolkit for ReQTL-specific data transformation (https://github.com/HorvathLab/ReQTL). In addition, we apply and compare two parallel strategies to correct for allele-mapping bias, known to affect VAF_RNA_ estimation: mapping to an SNV-containing index using HISAT2 (Kim *et al.*, 2015), and removal of reads mapped ambiguously after re-mapping with the alternative allele (WASP, Van de Geijn *et al.*, 2015). Finally, we systematically compare ReQTL and eQTL analyses performed on the same datasets, and analyze the subsets of variants identified by both and exclusively by either of the methods.

## 2 Materials and methods

### 2.1 Samples

The data and analyses presented in the current publication are based on the use of study data downloaded from the dbGaP web site, under dbGaP accession phs000424.v7.p2 (Genotype-Tissue Expression, GTEx). A total of 659 raw RNA-seq datasets from three different body sites—Nerve–Tibial (NT, 197 samples), Skin-Exposed, (SkE, 243 samples) and Skin-Non-Exposed (SkN, 216 samples)—were downloaded on 06/10/18 (Supplementary Table S1). The samples were selected based on the availability of directly estimated genotypes (for eQTL comparisons). All the RNA-seq libraries were generated using non-strand specific, polyA-based Illumina TruSeq protocol and sequenced to a median depth of 78 million 76-bp paired-end reads. The selection of tissue types was based on the availability of more than 150 samples with genotypes, and consideration for assessment of both distinct (NT versus Skin) and related (SkE versus SkN) tissue types.

### 2.2 RNA-seq data processing

**Fig. 2.**
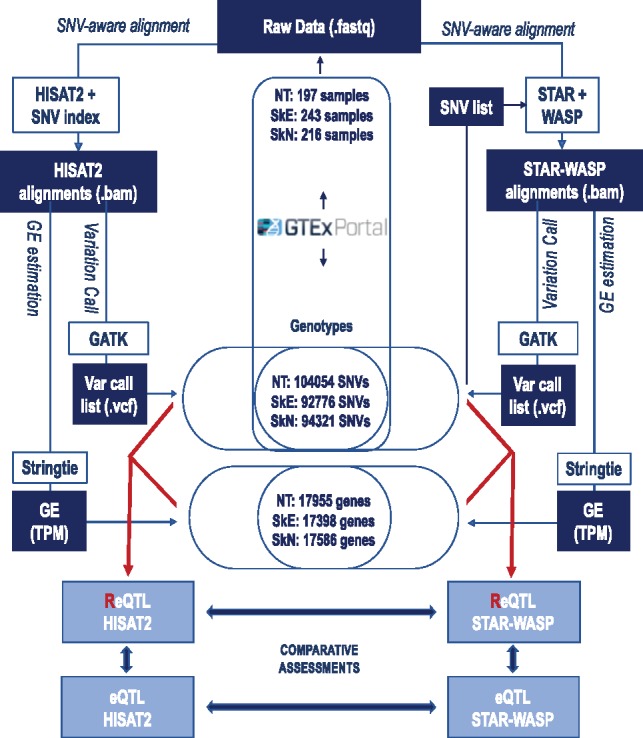
Approach to select input SNV loci and genes and comparative analyses between SNV-aware alignment strategies and ReQTL versus eQTL

SNV-aware alignment was performed using two strategies in parallel: (1) HISAT2 with an SNV index (Kim *et al.*, 2015), and (2) STAR alignment (Dobin, *et al.*, 2013) followed by removal of ambiguously aligned reads using WASP (Van de Geijn *et al.*, 2015). The alignments were processed downstream in parallel, and identical sets of genes and SNVs were used for between-pipelines comparative analyses (Fig. 2).

#### 2.2.1 Alignment using HISAT2 with SNV index

RNA-seq reads were aligned to the latest release of the human reference genome (hg38/GRCh38, Dec 2013) using HISAT2 (v. 2.1.0) with a SNV and transcript annotation index (Kim *et al.*, 2015). The SNV index was pre-built using DbSNP 144, and downloaded from the HISAT2 reference repository; (https://ccb.jhu.edu/software/-hisat2/index.shtml). The generated alignments were sorted by coordinates, indexed (Li *et al.*, 2009) and used for estimation of both gene expression (GE) and variant calling with subsequent VAF_RNA_ assessment.

#### 2.2.2 Alignment using STAR-WASP pipeline

First, we aligned the RNA-seq reads to GRCh38, using STAR v.2.6.1c in 2-pass mode with transcript annotations from assembly GRCh38.79. We called SNVs on the alignments (see below) and combined the SNVs called across all samples from a tissue type into a list of unique SNV positions. This list was then used as an input to WASP (Van de Geijn *et al.*, 2015) to test for allele mapping bias and to remove reads with ambiguous mapping due to an SNV. The generated alignments were processed for GE and VAF estimation in parallel with the HISAT2-generated alignments.

#### 2.2.3 Variant call

To call variants from RNA-seq data we used GATK (v. 4.0.8.0) and followed the provided best practices (Van der Auwera *et al.*, 2013). Briefly, we first marked duplicates to clean the data, then used the module SplitNCigarReads to reformat intron-spanning reads, followed by Base Quality Recalibration to re-adjust the base quality values. The datasets were then subjected to variant calling using the module HaplotypeCaller. Indel calls, and mitochondrial and contig variants were filtered-out. Using this pipeline, we called between 214 043 and 685 959 (average 355 201) SNVs in the individual samples from the HISAT2 alignments, and between 225 117 and 716 640 (average 371 610) from the STAR-WASP alignments. To retain high-quality SNV calls, we applied the VariantFiltration GATK module using as hard filters QUAL (Phred quality score) >100 and MQ (mapping quality) >60, and combined the filtered SNVs into a list of unique SNV positions per tissue (HISAT2/STAR-WASP: NT—1 038 361/1 204 315, SkE—950 858/1 076 441, SkN—932 665/966 812). After annotation (SeattleSeq v.14, DbSNP151), we retained SNVs present in the HISAT2 index, positioned outside repetitive regions, and with genotypes available from GTEx. These SNV lists were used for WASP re-alignment (see above) and for VAF_RNA_ estimation and subsequent ReQTL and eQTL analyses.

#### 2.2.4 Variant allele fraction (VAF_RNA_) estimation

Within a tissue type, we estimated n_var_ and n_ref_ and computed VAF_RNA_ for each of the positions in the list in each of the individual samples using the module readCounts previously developed in our lab (http://github.com/HorvathLab/NGS/tree/-master/readCounts; Movassagh *et al.*, 2016). Briefly, readCounts employs the pysam Python module to assess the read counts at every SNV position of interest in each of the alignments (samples) from a studied group (i.e. tissue). ReadCounts then filters aligned reads based on alignment quality metrics including length, gaps and mapping quality, and categorizes the remaining reads as having either the reference or the variant nucleotide. For ReQTL analyses, we retained only positions covered by a minimum of 10 total sequencing reads (ReQTL-fit VAF_RNA_); samples with VAF_RNA_ estimated from < 10 reads were assigned NA in the input matrices. Additionally, we excluded SNV positions with a monoallelic or missing (NA) signal in more than 80% of the samples from each tissue.

#### 2.2.5 Gene expression estimation

Gene expression was estimated from the alignments using Stringtie (version 1.3.4.) (Kim *et al.*, 2015; Pertea *et al.*, 2016), and TPM (transcripts per million) values were used for the ReQTL analyses. Pseudogenes were identified based on ensembl annotations (https://useast.ensembl.org/-info/data/biomart/index.html), and excluded from the analysis. Furthermore, within each tissue, we filtered out genes with a TPM value <1 in more than 80% of the samples. The TPM distribution was quantile-transformed using the average empirical distribution observed across all samples in the corresponding tissue (Aguet *et al.*, 2017). The effects of unobserved confounding variables on gene expression were quantified using probabilistic estimation of expression residuals (PEER), with 25 PEER factors (Stegle *et al.*, 2012).

#### 2.2.6 eQTL analyses

We performed eQTL for comparative analysis with ReQTL, using HISAT2 and STAR-WASP pipelines in parallel. The genotypes for each individual were obtained from DbGaP (phs000424.v7.p2), and the gene expression data, covariates and regression model were same as those used for the ReQTL analyses (see below). Following Aguet *et al.*, we identified significant associations after *P*-value correction using false discovery rate of 5% for the *cis*-associations, and 10% for the *trans*-associations.

## 3 Results

### 3.1 ReQTL approach

The overall approach for ReQTL analyses and comparative assessments is presented in Figure 2. We performed the ReQTL analyses separately for the three tissues, using a linear regression model as implemented in the package Matrix eQTL (Shabalin, 2012). Lists of SNV loci were generated based on the combined variation calls in each tissue after filtering for quality and position in repetitive regions. In addition, loci covered by fewer than 10 sequencing reads or with a monoallelic signal in more than 80% of the samples were excluded from the analyses. For direct comparisons between the HISAT2 and STAR-WASP pipelines, and with the eQTLs, we used the same input lists of SNV loci per tissue, which were generated based on: (i) accessibility for ReQTL analysis (as described above), (ii) presence in the pre-built HISAT2 SNV index and, (iii) availability of genotypes from the GTEx portal. This resulted in 104 054, 92 776 and 94 321 SNVs for the NT, SkE and SkN, respectively.

Similarly, for all ReQTL and eQTL analyses, we used the same input gene lists selected based on expression value above 1 TPM estimated from both HISAT2 and STAR-WASP alignments in at least 20% of the samples per tissue. This resulted in 17 955, 17 398 and 17 586 genes for the NT, SkE and SkN, respectively (Fig. 2). To account for covariates, we corrected for the top 25 PEER factors (Stegle *et al.*, 2012), reported race, sex and the top three VAF_RNA_ or genotype principal components (PCs), for ReQTL and eQTL, respectively. To be considered *cis*-ReQTL, a variant was required to reside within 1 megabase of the transcription start site of a gene. We retained for further analysis significant *cis*-associations using a false discovery rate cutoff of 5% (FDR < 0.05); to allow for direct comparison with the eQTL reported by Aguet *et al.*, (2017), for *trans*-associations we used an FDR cutoff of 10%.

### 3.2 Overall ReQTL findings

The numbers of significant *cis*- and *trans*-ReQTL correlations identified using HISAT2 and STAR-WASP pipelines in the individual tissues are shown in Table 1.

**Table 1.** Total and shared number of ReQTLs identified in each tissue

Tissue	Number ReQTL		Shared ReQTLs		
	HISAT2	**STAR**-**WASP**	Total N	% HISAT2	**% STAR**-**WASP**
***Cis***					
**NT**	19 602	30 623	15 660	79.8	51.1
**SkE**	17 239	24 776	13 324	77.3	53.8
**SkN**	13 161	19 897	10 346	78.6	52
***trans***					
**NT**	262	301	159	60.7	52.8
**SkE**	267	406	137	51.3	33.7
**SkN**	369	490	220	59.6	44.9

*Note:* The percentage values indicate the proportion of shared correlations out of the total number identified with the corresponding approach.

**Fig. 3.**
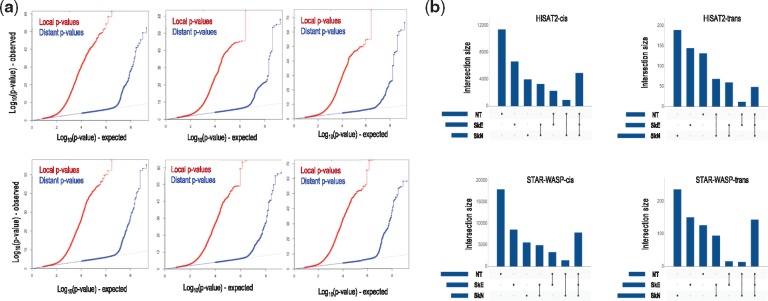
(**a**) QQ-plots of the ReQTL *P*-values: from left to right: NT, SkE, SkN, top: HISAT2 pipeline, bottom: STAR-WASP pipeline. (**b**) Relative representation of tissue-specific and shared ReQTLs. On each graph, the three plots on the left represent exclusive NT, SkE and SkN, ReQTLs, respectively; the 3-tissue overlapping ReQTLs are shown on the most-right

Across the three tissues, ReQTL analyses identified 33 596 significant *cis-* and 658 significant *trans*-correlations using the HISAT2 pipeline (Supplementary Table S2). The *cis*-correlations were composed of a total of 20 804 SNV loci and 5882 genes, while the *trans*-correlations involved 382 SNV loci and 316 genes. When using STAR-WASP alignments, ReQTL analysis resulted in a comparatively higher number of significant findings: 47 954 *cis*- and 784 *trans*-correlations (Supplementary Table S3). The *cis*-correlations included 27 873 SNV-loci and 7870 genes and the *trans*-correlations included 493 SNV loci and 337 genes. Quantile-quantile (QQ) plots are shown in Figure 3a, and shared and tissue-specific ReQTLs are presented in Figure 3b. Percent explained variation by the top 10 PCs for VAF_RNA_ and genotypes is shown in Supplementary Figure S1.

**Fig. 4.**
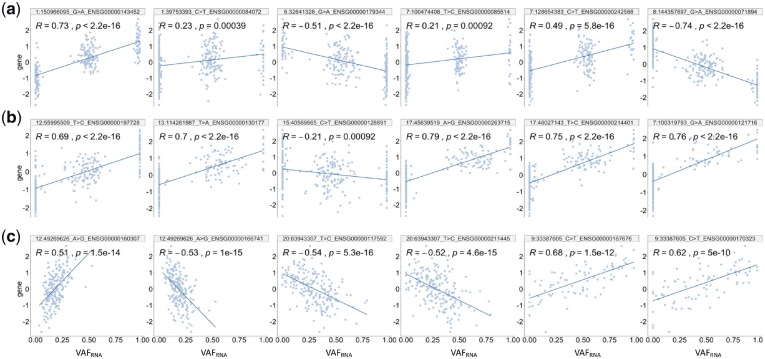
Correlation patterns identified by ReQTL analyses. (**a**) *cis*-ReQTL eQTL-like patterns. (**b**) *cis*-ReQTL patterns with non-extreme VAF_RNA_ values spread along the regression line. (**c**) *Trans*-ReQTLs—a characteristic pattern with most of the VAF_RNA_ values spread along the regression line

Representative examples of ReQTL are shown in Figure 4. In the *cis*-ReQTLs, we observed two major types of correlation patterns: eQTL-like, where the distribution of VAF_RNA_ values resembled the genotype distribution (Fig. 4a), and patterns where the intermediate VAF_RNA_ values are spread along the regression line (Fig. 4b). In the *trans*-ReQTLs, typical patterns had most of the VAF_RNA_ values spread along the regression line (Fig. 4c).

## 4 Comparative analyses

We assessed the differences between the ReQTLs produced through HISAT2 and STAR-WASP aligners and evaluated the proportion of eQTLs identifiable through ReQTL analysis. To do this, we performed eQTL analysis on the same input datasets of genes, SNV loci and covariates, replacing VAF_RNA_ with genotypes obtained from GTEx. In each tissue, we analyzed overlapping and exclusive ReQTL and eQTL outputs, as well as differences between the HISAT2 and STAR-WASP pipelines.

### 4.1 HISAT2 versus STAR-WASP ReQTLs

We first assessed the differences between ReQTLs called in HISAT2 and STAR-WASP alignments. For the *cis*-ReQTLs, a higher number of significant correlations was called using the STAR-WASP alignments (1.56-, 1.43- and 1.51- fold increase for the NT, SkE and SkN, respectively). The *cis*-ReQTLs identified by both pipelines represented more than 75% of all *cis* -ReQTLs called in the HISAT2 alignments, and a little over 50% of the *cis*-ReQTLs called in the STAR-alignments (Table 1). *Trans*-ReQTLs were found in substantially lower numbers, and showed a lower overall rate of agreement between HISAT2 and STAR-WASP.

To estimate the contribution of VAF_RNA_ and GE to the differences in the ReQTLs between the two approaches, we assessed the relative differences of VAF_RNA_ and GE estimated from HISAT2- and STAR-WASP alignments. To do this, we performed min-max scaling on the VAF_RNA_ and GE values separately to bring the values into the same numeric range. We then computed the absolute difference between VAF_RNA_ values estimated in each variant from the two alignments and compared to the corresponding differences in the GE estimation. This assessment showed a greater median difference between the STAR and HISAT GE values as opposed to VAF_RNA_. (*P* < 10e^−22^, Wilcoxon rank sum test, Supplementary Fig. S2), suggesting a larger contribution of GE to the differences in the two ReQTL estimations (See 4.2 below). This is also consistent with the very similar pattern observed in the comparative eQTL analyses between HISAT2 and STAR-WASP, where the only difference between the inputs is the GE estimation. Differences in GE estimation between alignments, including HISAT2 and STAR, are acknowledged and analyzed elsewhere (Baruzzo *et al.*, 2017; Raplee *et al.*, 2019). We note that the size of the STAR-WASP alignments was on average 32% larger than the corresponding HISAT2 alignment. Notably, VAF_RNA_ estimations from the two alignments were generally consistent, with the HISAT2 VAF_RNA_ values showing slightly higher variance (Supplementary Fig. S3).

### 4.2 eQTL-ReQTL exclusive and overlapping correlations

#### 4.2.1 Cis-correlations

For direct comparisons with eQTL analyses, the three genotypes corresponding to homozygous reference, heterozygous and homozygous variant genotype (0, 1 and 2, respectively) were converted to 0, 0.5 and 1. To parallel the ReQTL analyses, we first assessed the differences between HISAT2 and STAR-WASP eQTLs. While the absolute numbers of significant *cis*-eQTLs were higher than the ReQTLs, we observed a strikingly similar overlap between the HISAT2 and STAR-WASP eQTL calls (Supplementary Table S4). For the *cis*-eQTLs, the STAR-WASP pipeline produced a 1.54-, 1.39- and 1.51- fold greater number of significant correlations for the NT, SkE and SkN, respectively).

We next analyzed the proportion of shared and exclusive *cis*- ReQTLs and eQTLs (Table 2). The correlations called by both methods represented between 89 and 91% of all *cis*-ReQTL significant calls, and between 58.4 and 62.5% of the significant *cis*-eQTLs. Accordingly, in a side-by-side setting, up to a half of the *cis*-eQTLs are not called significant through ReQTL analyses, while approximately 10% of the significant *cis*-ReQTL correlations are not called through eQTL analyses. The percentage of eQTL-genes captured by ReQTL was between 72 and 78%, indicating that ReQTLs capture on average three quarters of the genetically regulated gene expression in transcribed regions.

**Table 2.** Total and shared number of cis-ReQTLs and eQTLs identified in each tissue

Tissue	Correlations		Shared ReQTLs-eQTLs		
	(HISAT2/STAR-WASP)		(HISAT2/STAR-WASP)		
	ReQTL	eQTL	Total N	% ReQTL	% eQTL
**NT**	19 602/30 623	29 553/45 556	17 681/27 870	90.2/91	59.8/61.2
**SkE**	17 239/24 776	25 245/35 311	15 338/22 086	89.0/89.1	60.8/62.5
**SkN**	13 161/19 897	20 285/30 475	11 828/18 069	89.9/90.8	58.4/59.3
**Genes**					
**NT**	3582/5157	4187/5878	3113/4586	86.9/88.9	**74.3/78.0**
**SkE**	3208/4417	3652/4878	2772/3804	86.4/86.1	**75.9/78.0**
**SkN**	2652/3729	3164/4364	2280/3257	85.9/87.3	**72.0/74.6**

Our analysis shows that a major contributor to the lower number of significant ReQTLs (as compared to eQTLs) is the lower proportion of VAF_RNA_ values (relative to the number of genotype values) available for each SNV locus in the samples from the studied samples. As mentioned above, for all of our analyses, we used the same lists of SNV loci to satisfy the requirement to have at least 20% samples from the studied tissue with VAR_RNA_ (non-NA) estimated from a minimum of 10 sequencing reads. Indeed, while all of the loci satisfied the 20% threshold, the actual percentage of samples with ReQTL-fit VAF_RNA_ estimation was lower than the samples with genotypes. Specifically, genotypes for each SNV were present in more than 99.9% of the samples, while VAF_RNA_ values were present on average in between 66.9 and 69.6% of the samples for each locus. Related to that, only up to 20% of the SNVs had VAF_RNA_ estimations in all samples per group, compared to above 97% for the genotypes (Supplementary Fig. S4).

In addition, we analyzed the concordance between genotypes and VAF_RNA_. To do this, we directly compared homozygous genotypes to monoallelic VAF_RNA_ calls, and heterozygous GTs to biallelic VAF_RNA_ calls (Supplementary Fig. S5). The heterozygous GTs and the biallelic VAF_RNA_ calls were concordant in nearly all of the samples, while the homozygous GTs had complete concordance in over 85% of the samples, and, for the over 90% of the discordant positions—within 10% difference. Further analysis showed that the discordant positions are largely overlapping between the three tissues and typically include loci covered by over 50 reads (with one or two sequencing reads bearing the discordant nucleotide). The mean deviation from the expected DNA genotype allele count was approximately 0.05 in all three tissue types (see Supplementary Fig. S5).

**Fig. 5.**
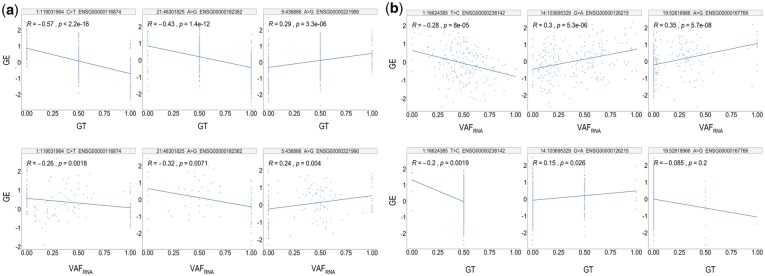
(**a**) eQTL-exclusive correlations (top) and their corresponding ReQTLs (bottom). The plots represent correlations from SkN. The available number of genotypes for the eQTL computation is 243 for all three SNVs (i.e. genotypes were available for all of the samples), while VAF_RNA_ values were present in 151 (62.1%) for the 1: 119031964_C > T locus, 186 (76.5%) for the 21: 46301825_A > C locus and 173 (71.2%) for the 5: 436866_A > G locus. (**b**) ReQTL-exclusive correlations (top) and their corresponding eQTLs (bottom). The examples show: (left) an SNV with a low number of homozygous genotype calls (1: 16624385_T > C, average heterozygosity in the human population: 0.494 ± 0.055); (middle): an SNV with relatively low number homozygous variant genotype calls (chr14: 103695329_G > A, average heterozygosity in the human population: 0.362 ± 0.224); (right) an SNV with relatively low number heterozygous and homozygous variant genotype calls (chr19: 52618966_A > G, average heterozygosity in the human population 0.294 ± 0.246). All the *P*-values are calculated based on the input for the plot and do not represent the ReQTL/eQTL FDR—corrected values

Examples of eQTL exclusive correlations, and their corresponding plots using the VAF_RNA_ from the same samples are shown in Figure 5a. For all three SNVs, genotypes were available for 100% of the samples in the particular tissue (SkE), while VAF_RNA_ values were present in between 62.1 and 76.5% of the samples.

On the other hand, ReQTL-exclusive correlations are frequently observed for SNVs where one or two of the genotypes are present in a low number of samples from the studied tissue (Fig. 5b). These cases include relatively rare SNVs, for which few samples from the dataset have heterozygous or homozygous variant genotypes, or common SNVs for which a predominant proportion of the samples have a heterozygous genotype.

In addition to direct ReQTL-eQTL comparisons, we assessed the overlap between ReQTL SNV loci called in our study and eQTL loci reported in the GTEx database (https://gtexportal.org/home-/V7). For the *cis*-comparisons, in each tissue, between 91.4 and 93% of the ReQTL loci were reported in GTEx (Table 3). The corresponding eQTL loci called in our study showed similar (and to a slight extend higher) overlap with GTEx eQTL loci. For both ReQTL and eQTL loci, these percentages were slightly higher for the loci called from the STAR-WASP alignments. We next estimated the proportion of GTEx SNVs called by our ReQTL analysis. The total number GTEx *cis*-SNVs participating in correlations with a *P*-value below 0.05 were 1 704 941, 1 635 959 and 1 520 254 for NT, SkE and SkN, respectively. From those, below 1% were present in the significant ReQTLs and eQTL outcomes from our study in any of the tissues (note that the number of input SNVs used for ReQTL was approximately 100 K for each tissue, see Fig. 2).

**Table 3.** Proportion of SNV loci participating in significant *cis*-correlations and reported in the GTEx database

Tissue	Alignment	Tissue	N_Loci	N Loci in GTEx	% Loci in GTEx
*Cis*					
ReQTL	HISAT2	NT	9177	8389	91.4%
		SkE	6650	6127	92.1%
		SkN	5990	5486	91.6%
	STAR-WASP	NT	13 227	12 275	92.5%
		SkE	9015	8388	93.0%
		SkN	8482	7845	92.5%
eQTL	HISAT2	NT	11 627	10 921	93.9%
		SkE	8089	7637	94.4%
		SkN	7512	7097	94.5%
	STAR-WASP	NT	16 511	15 618	94.6%
		SkE	10 592	10 053	94.9%
		SkN	10 281	9774	95.0%

#### 4.2.2 Trans-correlations

Using the described settings, our analysis identified between 262 and 490 *trans*-ReQTLs in the individual tissues (Table 4). Specifically, a total of 658 and 784 *trans*-ReQTLs were called from the HISAT2 and STAR-WASP alignments across the three studied tissues (See Supplementary Tables S2 and S3, respectively). In contrast to the *cis*-correlations, *trans*-ReQTLs and *trans*-eQTLs were identified in similar (and substantially lower) numbers in our study. The low number of *trans*-ReQTLs is expected given the known high tissue-specificity of *trans*-eQTLs, and the related confounding effects in bulk tissue samples with heterogeneous cellular composition (Westra *et al.*, 2013). For approximately half of ReQTLs in each tissue, the SNV was positioned on a different chromosome than the paired gene.

**Table 4.** Total and shared number of *trans*-ReQTLs and eQTLs identified in each tissue

Tissue	Correlations		Shared ReQTLs-eQTLs		
	(HISAT2/STAR-WASP)		(HISAT2/STAR-WASP)		
	ReQTL	eQTL	Total N	% ReQTL	% eQTL
**NT**	262/301	162/188	162/188	61.8/62.5	68.9/56.1
**SkE**	267/406	168/351	118/240	44.2/59.1	70.2/69.4
**SkN**	369/490	257/425	190/282	51.5/57.6	73.9/66.4
**Genes**					
**NT**	85/91	34/41	23/27	27/29.7	67.6/65.9
**SkE**	103/96	24/39	19/29	18.4/30.2	79.2/74.4
**SkN**	116/123	25/42	13/27	11.2/22.0	52.0/64.3
**SNVs**					
**NT**	172/184	185/243	135/156	78.5/84.8	73.0/84.8
**SkE**	136/274	133/296	97/209	71.3/76.3	72.9/70.1
**SkN**	216/293	218/353	168/238	77.8/81.2	77.1/67.4

The above findings are consistent with the GTEx eQTL analysis, where only 673 *trans*-eQTLs are found across 44 studied tissues (at FDR < 0.1), as compared to over 7 million *cis*-eQTLs (at FDR < 0.05). From the 673 *trans*-eQTLs in GTEx, only 3 were called in NT, 16 in SkE, and 1 in SkN. None of the SNVs participating in these 20 correlations satisfied the criteria for inclusion in ReQTL analysis.

To investigate the types of SNVs correlated with gene expression in a *trans* mode, we performed comparative gene ontology (GO) analysis of the genes bearing ReQTL- and eQTL-exclusive *trans*-acting SNVs, in the categories protein class and molecular function, (including at the level of transcription factor) using PANTHER classification system (http://pantherdb.org, Thomas *et al.*, 2006). This analysis showed largely similar patterns between the groups. In addition, we intersected the above *trans*-SNV bearing genes with the list of annotated long non-coding RNAs (https://lncipedia.org, Volders *et al.*, 2019), which revealed that up to 5% of the genes with *trans*-acting SNV in each of the groups are known lnc-RNAs. The lack of significant differences between *trans*-ReQTLs and QTLs is possibly due to the overall low number of *trans*-correlations.

To assess potential mechanisms of action of the *trans*-ReQTLs, we determined if their SNV loci also participated in significant *cis*-ReQTL correlations. In our data, 67 and 70% of the *trans*-SNVs (HISAT2 and STAR-WASP pipeline, respectively) were implicated in a significant *cis*-correlation at FDR < 0.05. This finding is similar to the GTEx *trans*-eQTLs and suggests that *trans*-ReQTLs frequently reflect gene-gene interactions, including those within their harboring gene.

## 5 Functional Re-QTL annotations

**Fig. 6.**
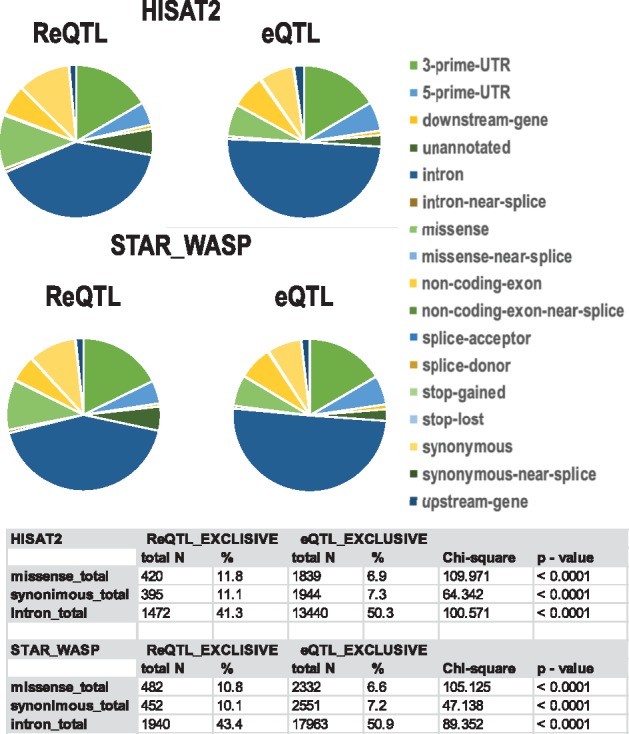
Distribution of functional annotations of SNVs participating in ReQTL-exclusive and eQTL-exclusive correlations. *missense_total: missense + missense-near-splice; synonymous_total = synonymous + synonymous-near-splice; intron_total = intron + intron-near-splice*

We also assessed the Re-QTL- and eQTL-exclusive SNV loci with respect to function, position and annotation, using the Variant Effect Predictor (VEP) https://www.ensembl.org/vep (Fig. 6). Due to the small number of ReQTL-exclusive findings, the distribution of functional annotations was assessed on the combined numbers of *cis*-acting SNVs across the three tissues. The two major annotation categories with significant differences in their distribution were exonic, which represented a higher proportion of ReQTL-exclusive SNVs (both synonymous and missense, separately analyzed), and intronic, which represented a higher proportion of eQTL exclusive SNVs (*P* < 0.001, chi-square test; all the differences were within 10%). In addition, we performed an analysis of the effect sizes in the above annotations. When all significant ReQTLs and eQTLs were analyzed, the mean and median values of the effect sizes were generally similar, with slightly higher effect sizes in the ReQTLs in most of the functional categories (Supplementary Fig. S6). This effect was stronger in the groups of ReQTL-exclusive and eQTL-exclusive SNVs (large annotation categories shown on Supplementary Fig. S7).

## 6 *Cis*- and *Trans*- ReQTL annotations

In the above results, for direct comparisons between eQTL and ReQTL, we distinguished *cis*- from *trans*-associations based on the commonly used in the eQTLs relative position of the SNV in regards to the gene chromosomal interval [measured as genetic distance in nucleotides (nt)]. In contrast to the DNA-estimated eQTLs, ReQTLs are assessed directly from transcripts. Accordingly, an alternative *cis*-annotation is based on the co-location of the SNV locus within the transcribed gene. To enable this annotation, we provide an option in the *run_matrix_ReQTL.R* script to perform the analysis without *cis/trans* annotation after which *annotate_cis_trans.R* can be run to annotate the significant correlations as described above.

## 7 ReQTL application

We note several considerations for the application of ReQTL analyses. First, because ReQTLs are based on VAF_RNA_, they are confined to expressed SNV loci in the studied sample-set and are not designed to capture variants in transcriptionally silent genomic regions. Related to that, SNV loci with low expression levels (below the required threshold for minimum number of RNA-seq reads) are not fit for ReQTL analyses. The threshold for minimum RNA-seq reads is critical for reliable estimation of VAF_RNA_. In our study, we have selected a threshold of 10 RNA-seq reads to determine positions suitable for ReQTL analysis, based on considerations for sequencing depth and confidence in the VAF_RNA_ assessment. Our experiments with various minimum thresholds show that higher thresholds increase the accuracy of the VAF_RNA_ estimation, but naturally retain a lower number of variants for analysis (Movassagh *et al.*, 2016). In the readCounts package (https://github.com/HorvathLab/NGS/tree/master/readCounts), this threshold is flexible and can be set at the desired level depending on the depth of sequencing and required confidence in the assessment of VAF_RNA_.

Second, even when SNVs are expressed and accessible for ReQTL analyses, ReQTL identifies a lower number of significant correlations as compared to eQTL. In a side-by-side application, ReQTL captures on average around 60% of the eQTL-identifiable correlations. Our analysis shows that this is mainly due to the fact that, for many loci, ReQTL VAF_RNA_ values are available for only a proportion of the samples (a minimum of 20% is used in this study), as compared to genotypes which are typically available for the vast majority of the studied samples. At the same time, due to the fact that ReQTLs typically capture multiple SNVs from the same gene, this method can identify a large proportion of the eQTL-identifiable genes (approximately three quarters in our analysis). This is mostly due to the fact that most of the ReQTL genes were significantly correlated with multiple SNVs, which largely agreed in regards to effect size and also showed strong concordance in VAF_RNA_ values (Supplementary Fig. S8). Regarding the above considerations, the proportions of ReQTL-identifiable correlations and genes are expected to increase with the sequencing depth of RNA-seq datasets.

Third, it is important to note that even when a genetically regulated gene is captured by ReQTL analysis, the SNVs correlated to this gene may not include the actual causative SNV, but its co-allelic (in linkage disequilibrium, LD) SNVs. This is particularly the case for regulatory SNVs positioned outside the gene transcribed region. While eQTL analyses also capture variants in LD with the actual causative variant, in the eQTLs this effect can be controlled for by using the genome-wide estimated effect sizes. In the ReQTLs, due to the restriction of the SNV input sets to transcribed regions, causality analyses require careful consideration of potentially missed co-allelic expression regulators.

On the other hand, ReQTL analyses identify about 10% more correlations in addition to those found through eQTL analyses. These include correlations where the significance of the eQTL association is diminished by asymmetric distribution of genotypes (Fig. 5b).

With respect to gene expression, ReQTL analysis can use the same data processing as is used for eQTLs, including adjustment for covariates. In this study, we closely followed the pipeline employed by the GTEx Consortium, correcting for reported race, sex and hidden confounders using the top 25 PEER factors based on sample size (Aguet *et al.*, 2017). In addition, we quantile-transformed the gene expression, as is customary in eQTL analyses. As a result, we observed a strong linear correlation between quantile-transformed, covariate-adjusted gene expression and VAR_RNA._ To fully explore ReQTLs, other expression-transformation strategies (Palowitch *et al.*, 2018) may also be applicable. In addition, the gene expression estimation is known to strongly depend on the RNA-seq alignment method (Baruzzo *et al.* 2017; Raplee *et al.*, 2019). In our study, we test two popular aligners—HISAT2 and STAR—which show substantial overlap, but also considerable differences in the ReQTL estimation (Table 1). Importantly, for ReQTL applications, the choice of aligner is also strongly related to the ability to confidently estimate VAF_RNA._ (See below). Our analysis shows that the differences in the ReQTL between the two approaches are driven mostly by the differences in the estimation of GE, while the VAF_RNA_ comparisons between paired samples were largely concordant (Supplementary Fig. S2).

VAF_RNA_ estimation can be also affected by technical parameters, the most important being allele mapping bias (Degner *et al.*, 2009). While shown to have little to no effect on gene expression estimation (Panousis *et al.*, 2014), mapping bias can lead to overestimation of the reference allele fraction (Brandt *et al.*, 2015). For ReQTLs, we applied the selected alignments in an SNV-aware setting. Specifically, HISAT2 was used with a genome-wide dbSNP index, and STAR-alignment was followed by removal of ambiguously mapped reads after checking for consistent mapping with the read containing the alternative nucleotide against a list of SNVs of interest. In our case, the list of SNVs of interest was generated by combining the variant call produced by GATK across all the samples from a tissue. We then systematically compared the outcomes. First, we did not observe significant signs of allele-mapping bias in either of the two outcomes (Figs 4 and 5), but bias was detectable in the ReQTL correlations when non-SNV-aware versions of the alignments were used. Second, the STAR-WASP pipeline produced a higher number of significant ReQTLs, as well as moderate, but consistently higher overlap with eQTL outcomes (Tables 2–4). On the other hand, the HISAT2 analysis included fewer steps and was significantly faster and more memory efficient.

Additional factors, including hidden confounders, can also impact the assessment of VAF_RNA_. To minimize such effects, we apply highly conservative settings to the alignment, variant calling and the read count assessment, correct for VAF_RNA_ PCs, and closely follow the best practices for data processing in allelic analysis (Castel *et al.*, 2015). In the presented results, we used the top 3 PCs to enable comparisons to eQTLs from the GTEx database. We have also tested ReQTL analyses with 5, 7 and 10 PCAs and observed that the number of significant ReQTLs slightly decreases with the number of PCs used.

Importantly, in contrast to the genotypes, VAF_RNA_ varies between different tissues and cell types, often due to tissue-specific regulatory mechanisms (Savova *et al.*, 2016). Furthermore, due to the dynamic nature of RNA transcription, it is expected that VAF_RNA_ (together with gene expression) will vary depending on conditions, disease state and random factors. Therefore, interpretation of ReQTL findings requires consideration of the dynamics of the correlation, similar to interpretation of gene expression.

For ReQTL applications, it is important to note that ReQTLs do not necessarily require prior variant calls and can be run on custom pre-defined lists of genomic positions such as those in dbSNP or a database of RNA-editing sites.

## 8 Discussion

Traditional eQTLs assess the number of variant-harboring alleles estimated from DNA data (N ∈ {0, 1, 2} for diploid genomes), in correlation with RNA-derived gene or transcript abundance across a population of individuals/samples. The recent advances in the approaches to infer genotypes form RNA-seq data have have enabled eQTL analyses using RNA-estimated genotypes (Tung *et al.*, 2015). While such approaches are confined to expressed SNV loci, they bring with them the benefit of using a single type of data (RNA-seq), which makes it possible to analyze large datasets across species and conditions, while reducing the costs and challenges associated with manipulating large volumes of data.

In our method—ReQTL—the genotypes are substituted for the VAF_RNA_ at expressed SNV loci; both VAF_RNA_ and the gene expression are assessed from the same sets of RNA-seq data. Compared to using the DNA-allele count, correlation of VAF_RNA_ with gene expression holds several technical advantages. First, as mentioned above, VAF_RNA_ constitutes a continuous measure and allows for precise quantitation of the allele representation. Second, since VAF_RNA_ and gene expression levels can be retrieved from a single source of transcriptome sequencing data alone, ReQTL analyses naturally avoid sample-specific and batch effects.

We envision several useful ReQTL applications with considerable potential to facilitate the discovery of novel molecular interactions. First, for sets where matched DNA is not available, ReQTL can be used to identify a subset of variation-expression relationships. However, it is important to note that ReQTL is not a direct replacement for eQTLs. Second, ReQTL can be applied to study regulatory SNVs, such as those residing in splicing factors binding sites, stop-codon altering SNVs and other motif-altering SNVs that are positioned in expressed regions. For the latter, we expect that ReQTL will be useful for assessing variants which alter motifs recognizable by RNA-binding molecules. Third, due to the continuous nature of VAF_RNA_, ReQTL can be used to study RNA-editing sites for which the VAF_RNA_ is highly variable (RNA-editing sites are excluded from the current analysis due to their position in repetitive genomic regions). Finally, there are a variety of potential future applications of ReQTL, including estimation of splicing QTLs from RNA-seq (i.e. RsQTL), and protein-level correlations (i.e. RpQTL).

## Funding

This work was supported by McCormick Genomic and Proteomic Center (MGPC), The George Washington University; [MGPC_PG2018 to AH] and UL1TR000075 from the NIH National Center for Advancing Translational Sciences (AH, KAC). Its contents are solely the responsibility of the authors and do not necessarily represent the official views of the National Center for Advancing Translational Sciences or the National Institutes of Health.

*Conflict of Interest*: none declared.

## Supplementary Material

btz750_Supplementary_Data
