# Supplementary material for: ReQTL: identifying correlations between expressed SNVs and gene expression using RNA-sequencing data
Source: Bioinformatics. 2019 Oct 7;36(5):1351–9. doi: 10.1093/bioinformatics/btz750 (PMC7058180; doi:10.1093/bioinformatics/btz750)
Supplement: btz750_Supplementary_Data [file btz750_supplementary_data.zip › btz750-Suppl_Data/S_Figure_1_PCs.pdf]

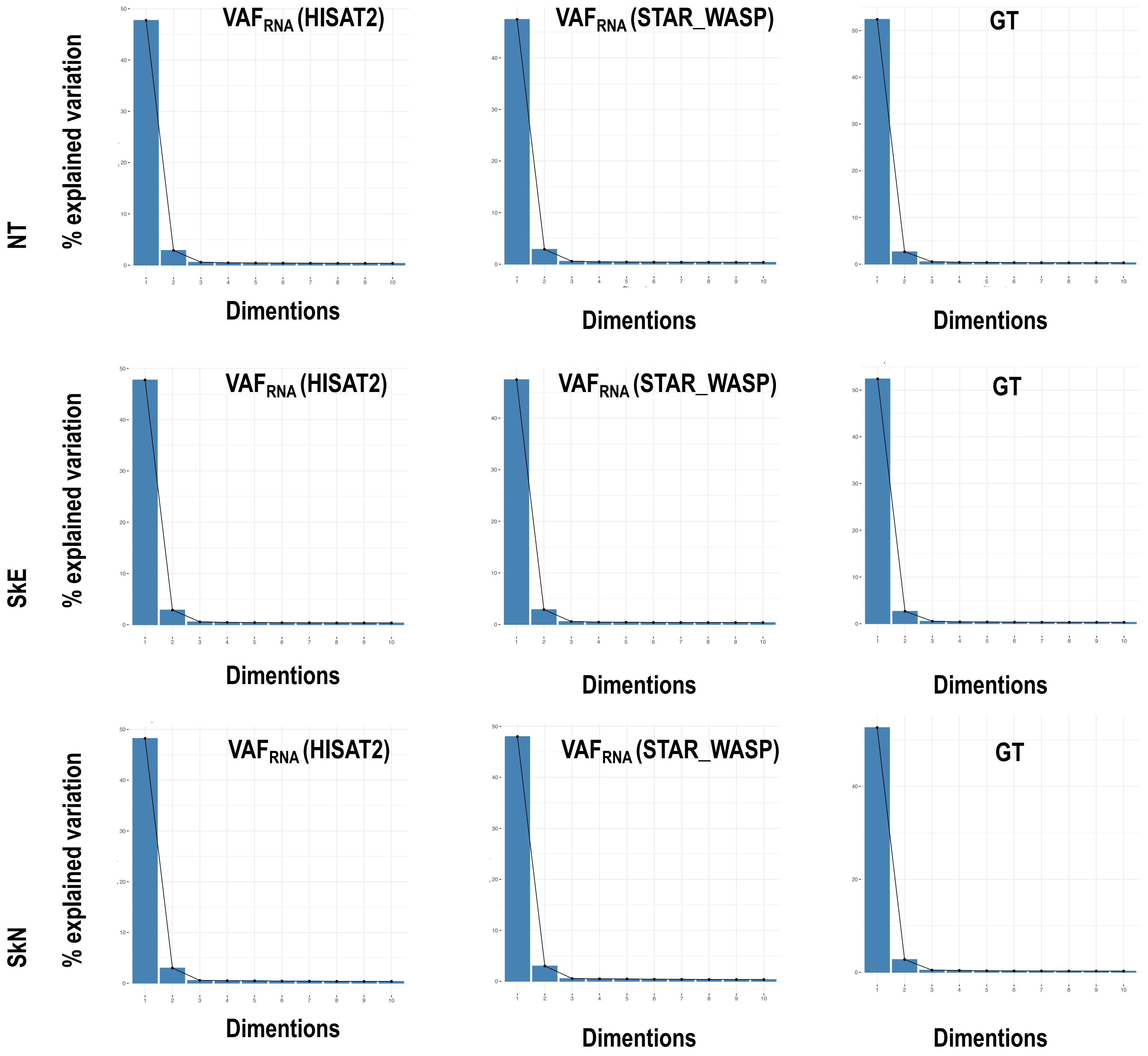

S_Figure_1. Percentage explained variance of the top 10 Principal Components (PC) for VAF$_{RNA}$ estimated from HISAT2 alignments (left), STAR_WASP alignments (middle) and Genotypes (GT, left). PCA shows that more than 50% of VAF$_{RNA}$ variance is explained affected by the top two PC; the observation is similar for the genotypes, where the top two PCAs explained roughly 60% of the variance. In the presented results we used the top 3 PCAs to enable comparisons to eQTLs from GTEx. We have also tested ReQTL analyses with 5, 7, and 10 PCAs, within which range we observed that the ReQTL number slightly decreases with the increased number PCAs.
