# Supplementary material for: ReQTL: identifying correlations between expressed SNVs and gene expression using RNA-sequencing data
Source: Bioinformatics. 2019 Oct 7;36(5):1351–9. doi: 10.1093/bioinformatics/btz750 (PMC7058180; doi:10.1093/bioinformatics/btz750)
Supplement: btz750_Supplementary_Data [file btz750_supplementary_data.zip › btz750-Suppl_Data/S_Figure_2_VAFvsGT.pdf]

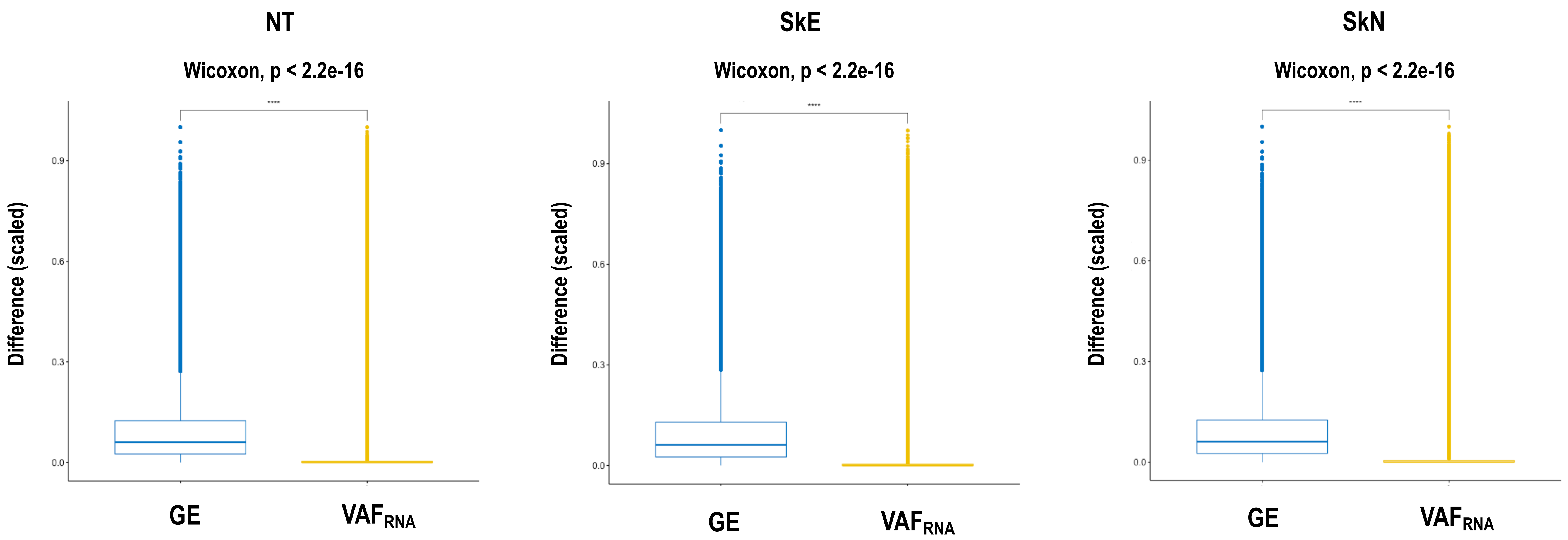

S_Figure_2. Difference between the gene expression (GE) and VAF_{RNA} values estimated in each sample from the HISAT2 and STAR_WASP alignments, estimated as absolute distance between the two estimations in the same samples, after scaling. Greater differences are observed between the GE estimations ($p < 10e-22$, Wilcoxon rank sum test for all three tissues), suggesting larger contribution of GE to the differences in the ReQTL estimations.
