# Supplementary material for: ReQTL: identifying correlations between expressed SNVs and gene expression using RNA-sequencing data
Source: Bioinformatics. 2019 Oct 7;36(5):1351–9. doi: 10.1093/bioinformatics/btz750 (PMC7058180; doi:10.1093/bioinformatics/btz750)
Supplement: btz750_Supplementary_Data [file btz750_supplementary_data.zip › btz750-Suppl_Data/S_Figure_3_Variance.pdf]

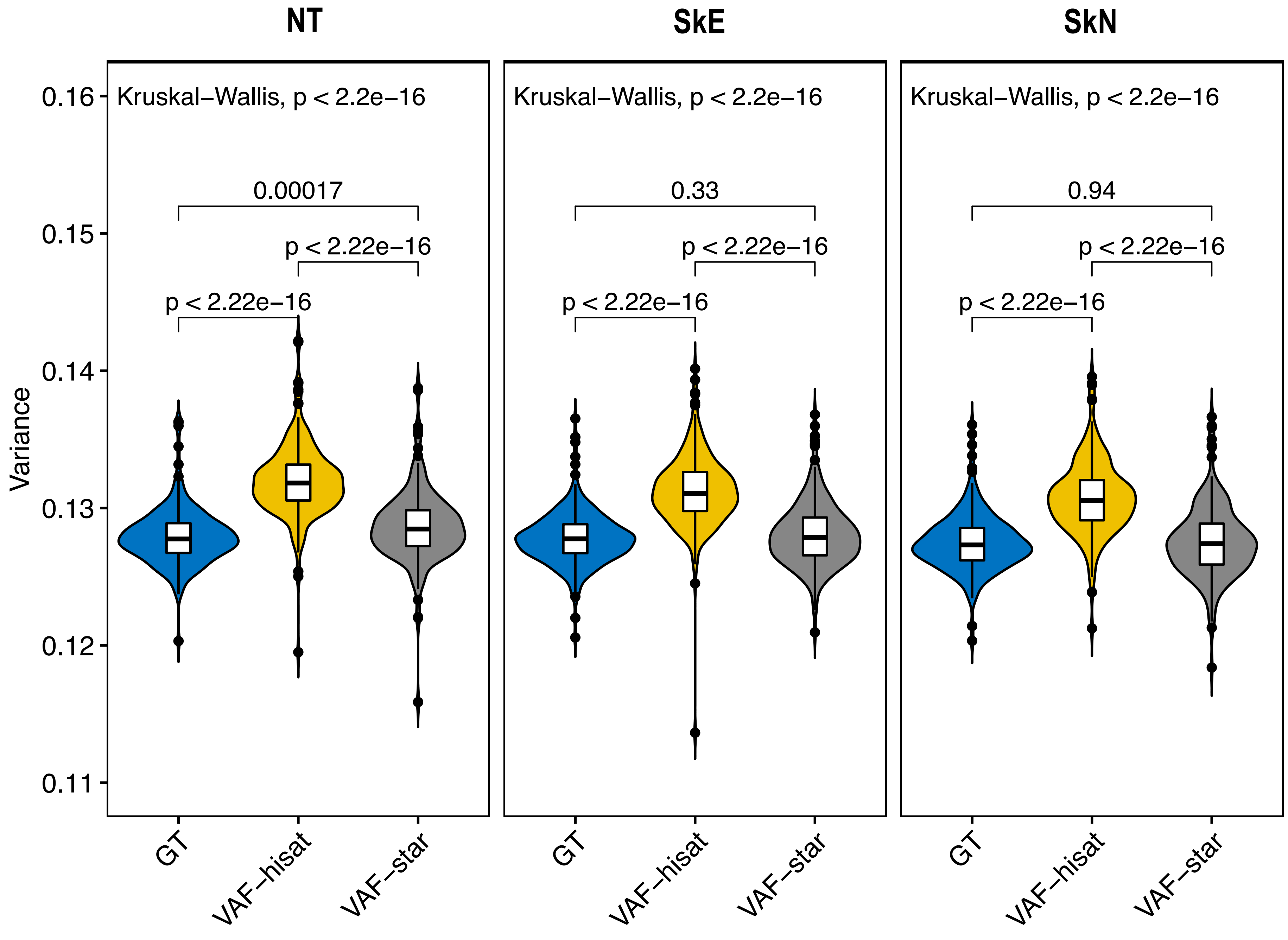

S_Figure_3. Variance of genotypes (GT), and VAF_{RNA} estimations from HISAT2 and STAR-WASP. The estimations were generally consistent, with the HISAT2 VAF_{RNA} values showing slightly higher (but still low) variance.
