# Supplementary material for: ReQTL: identifying correlations between expressed SNVs and gene expression using RNA-sequencing data
Source: Bioinformatics. 2019 Oct 7;36(5):1351–9. doi: 10.1093/bioinformatics/btz750 (PMC7058180; doi:10.1093/bioinformatics/btz750)
Supplement: btz750_Supplementary_Data [file btz750_supplementary_data.zip › btz750-Suppl_Data/S_Figure_4_Perc_samples with GT and VAF.pdf]

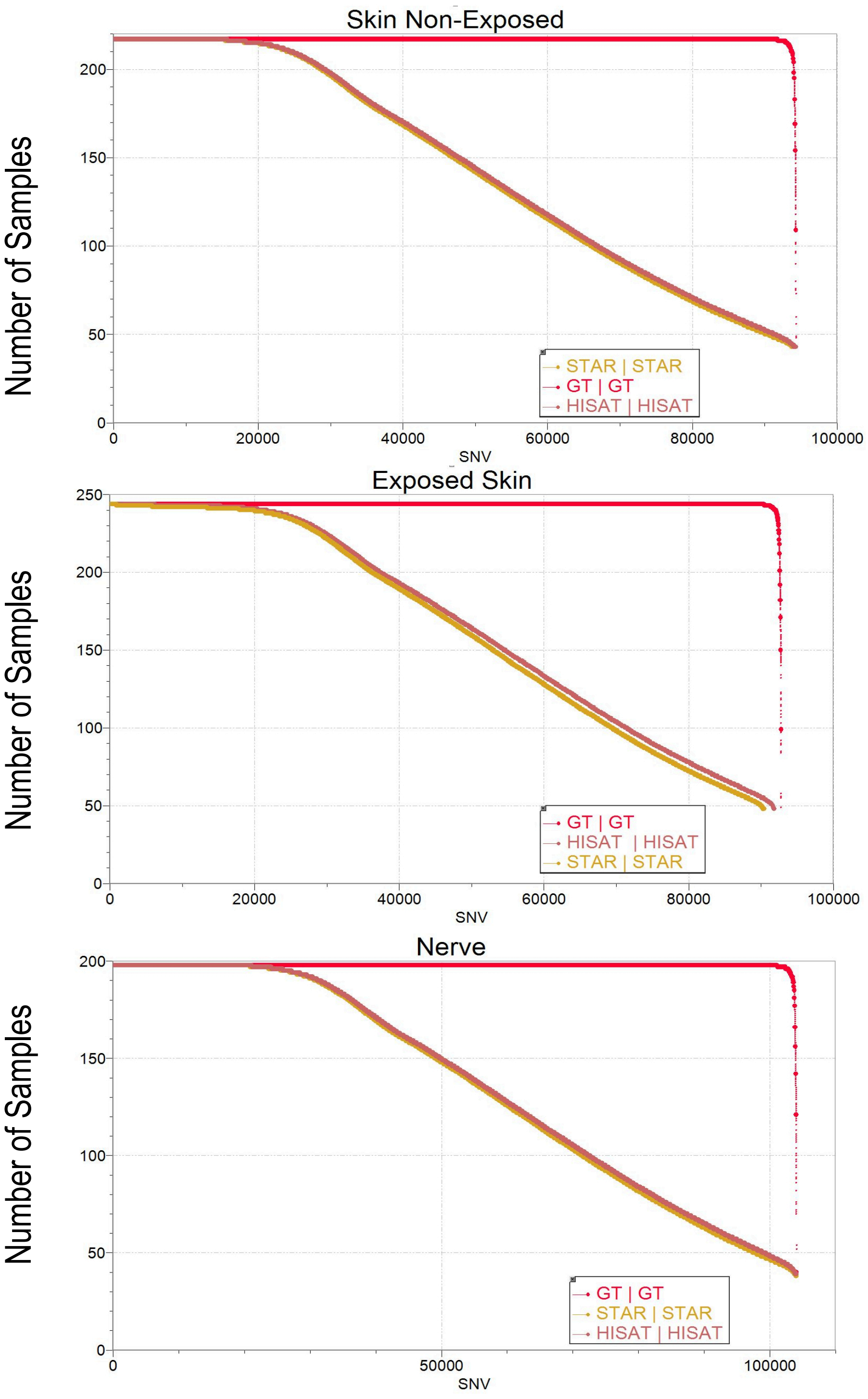

S_Figure_4. Percentage of samples with non-NA value in the genotype (red), HISAT2 (purple) and STAR_WASP (orange) input matrices. We required at least 20% of the samples to have VAF_{RNA} or GT for the locus to be assessed for ReQTL or eQTL, respectively. The actual percentage of samples with VAF_{RNA} estimation was lower than the samples with genotypes: GT values for each SNV were present in more than 99.9% of the samples, while VAF_{RNA} values were present on average in between 66.9% and 69.6% of the samples for each locus. Related to that, only up to 20 % of the SNVs had VAF_{RNA} estimations in all samples per group, compared to above 97% for the genotypes.
