# Supplementary material for: ReQTL: identifying correlations between expressed SNVs and gene expression using RNA-sequencing data
Source: Bioinformatics. 2019 Oct 7;36(5):1351–9. doi: 10.1093/bioinformatics/btz750 (PMC7058180; doi:10.1093/bioinformatics/btz750)
Supplement: btz750_Supplementary_Data [file btz750_supplementary_data.zip › btz750-Suppl_Data/S_Figure_5_VAF-GT_agreement.pdf]

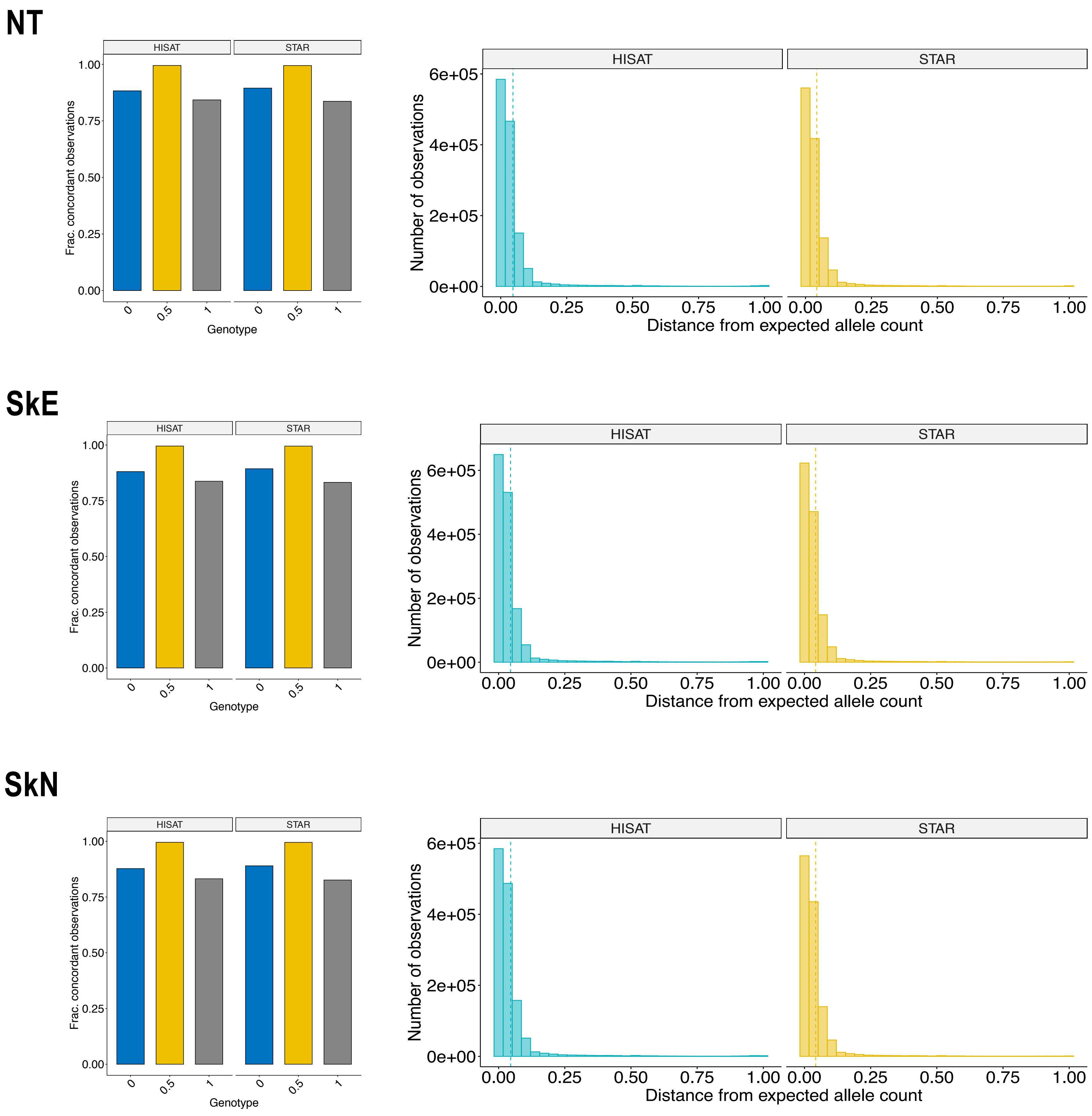

S_Figure 5. VAF_{RNA} – Genotype agreement. The heterozygote GT largely agreed with biallelic VAF_{RNA}. Disagreement was seen in up to 15% of the homozygote genotypes. From the disagreeing GT and VAF_{RNA}, more than 50% showed distance from the expected genotype below 0.05 (i.e GT = 1, VAF_{RNA} = 0.95, or GT = 0, VAF_{RNA} = 0.05). The results across the three tissues were highly concordant.
