# Supplementary material for: ReQTL: identifying correlations between expressed SNVs and gene expression using RNA-sequencing data
Source: Bioinformatics. 2019 Oct 7;36(5):1351–9. doi: 10.1093/bioinformatics/btz750 (PMC7058180; doi:10.1093/bioinformatics/btz750)
Supplement: btz750_Supplementary_Data [file btz750_supplementary_data.zip › btz750-Suppl_Data/S_Figure_6_Effect_sizes_all.pdf]

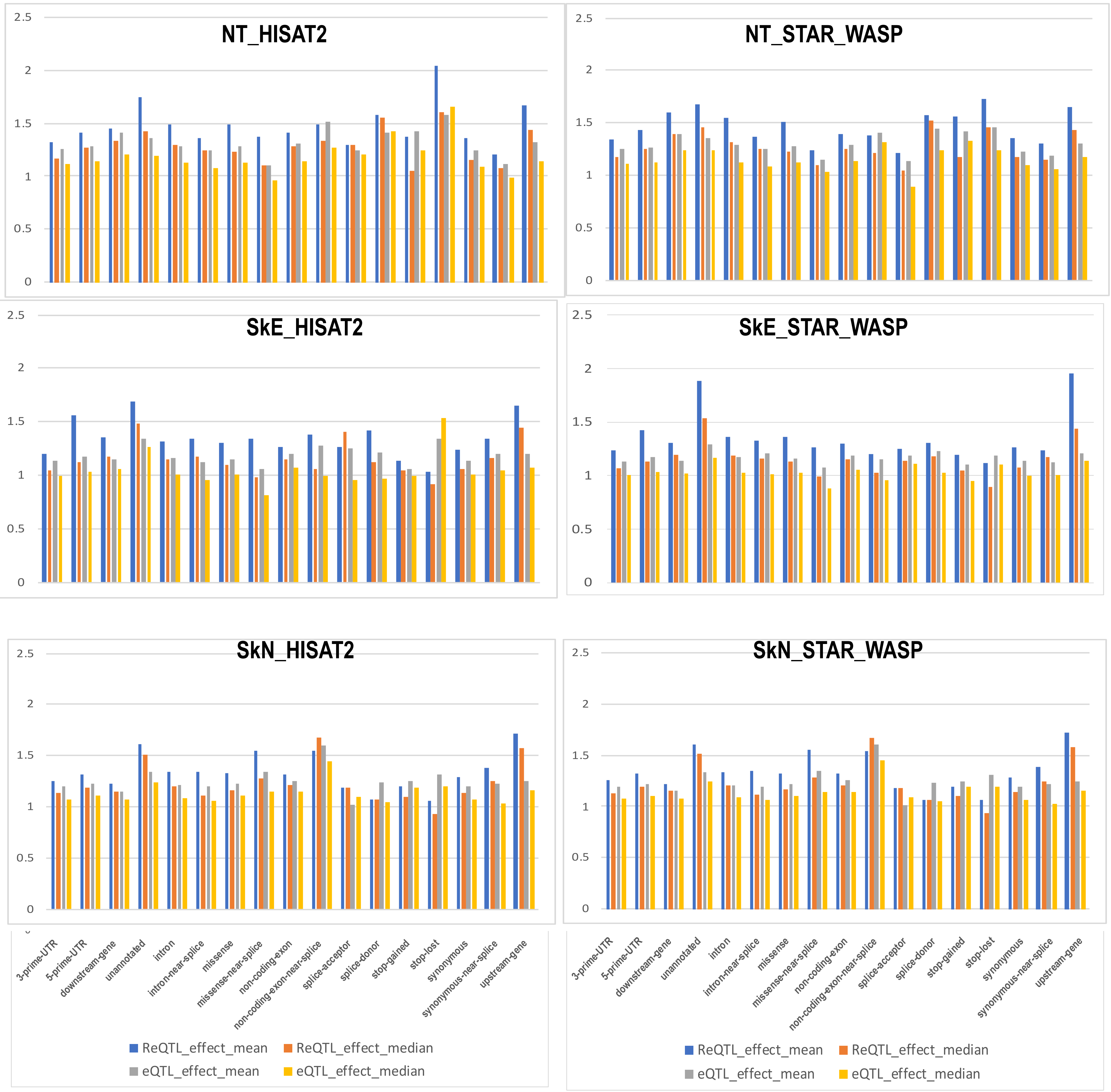

S_Figure 6. Effect sizes for SNVs participating in significant cis-ReQTL and cis-eQTLs by functional (position in the gene) annotation: Mean and Median Values. For most of the functional categories, a slightly higher effect sizes were estimated for the ReQTL SNVs.
