# Supplementary material for: ReQTL: identifying correlations between expressed SNVs and gene expression using RNA-sequencing data
Source: Bioinformatics. 2019 Oct 7;36(5):1351–9. doi: 10.1093/bioinformatics/btz750 (PMC7058180; doi:10.1093/bioinformatics/btz750)
Supplement: btz750_Supplementary_Data [file btz750_supplementary_data.zip › btz750-Suppl_Data/S_Figure_7_Effect_sizes_exclusive.pdf]

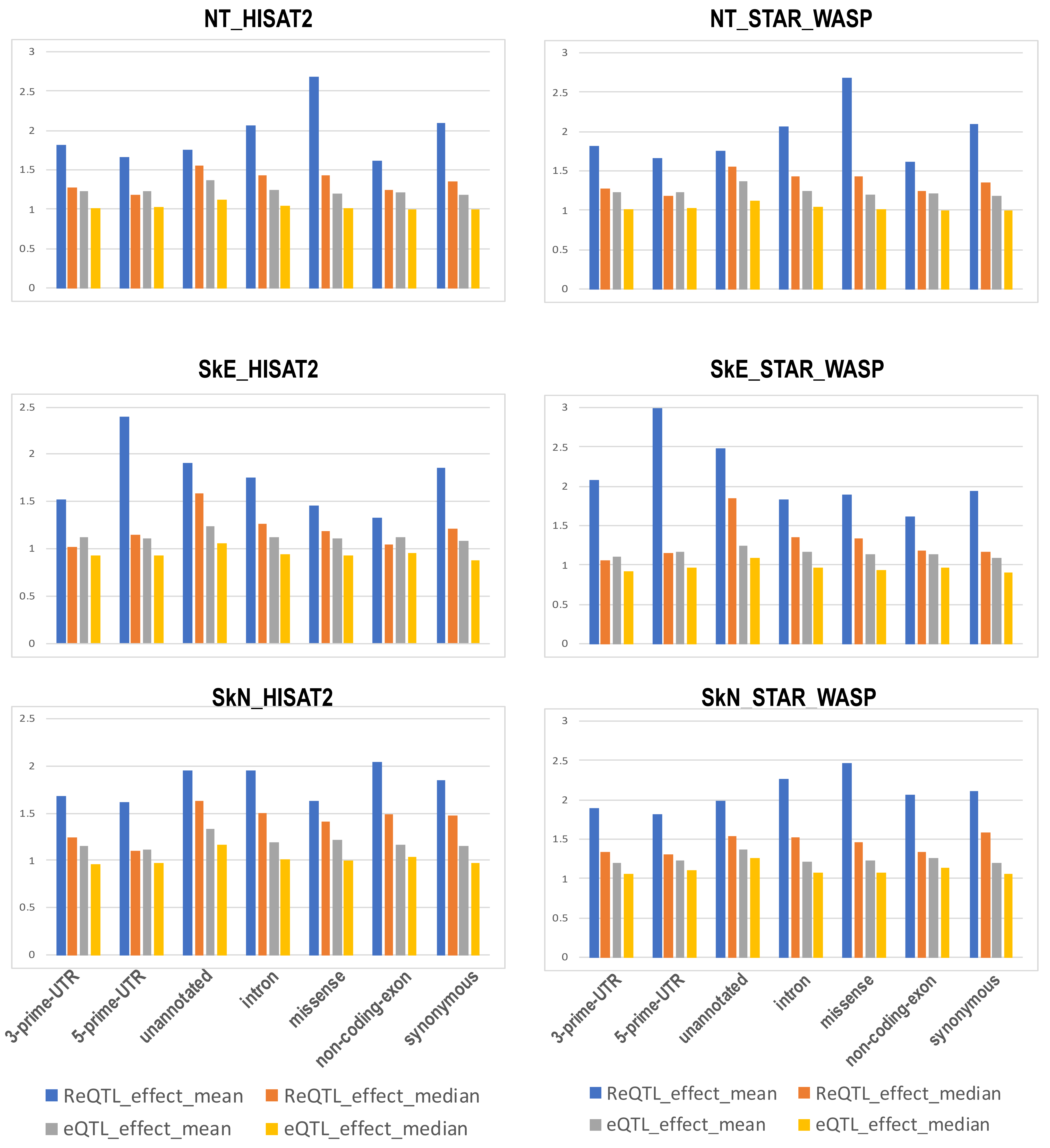

S_Figure 7. Effect sizes for SNVs participating exclusively in ReQTL or eQTLs cis-correlations by functional (position in the gene) annotation: mean and median values; only the largest categories are displayed due to small number of SNVs in the rest. Higher effect sizes are found for SNVs participating exclusively in ReQTL correlations as compared to the eQTL-exclusive SNVs.
