# Supplementary material for: ReQTL: identifying correlations between expressed SNVs and gene expression using RNA-sequencing data
Source: Bioinformatics. 2019 Oct 7;36(5):1351–9. doi: 10.1093/bioinformatics/btz750 (PMC7058180; doi:10.1093/bioinformatics/btz750)
Supplement: btz750_Supplementary_Data [file btz750_supplementary_data.zip › btz750-Suppl_Data/S_Figure_8_VAF-VAF_correlations.pdf]

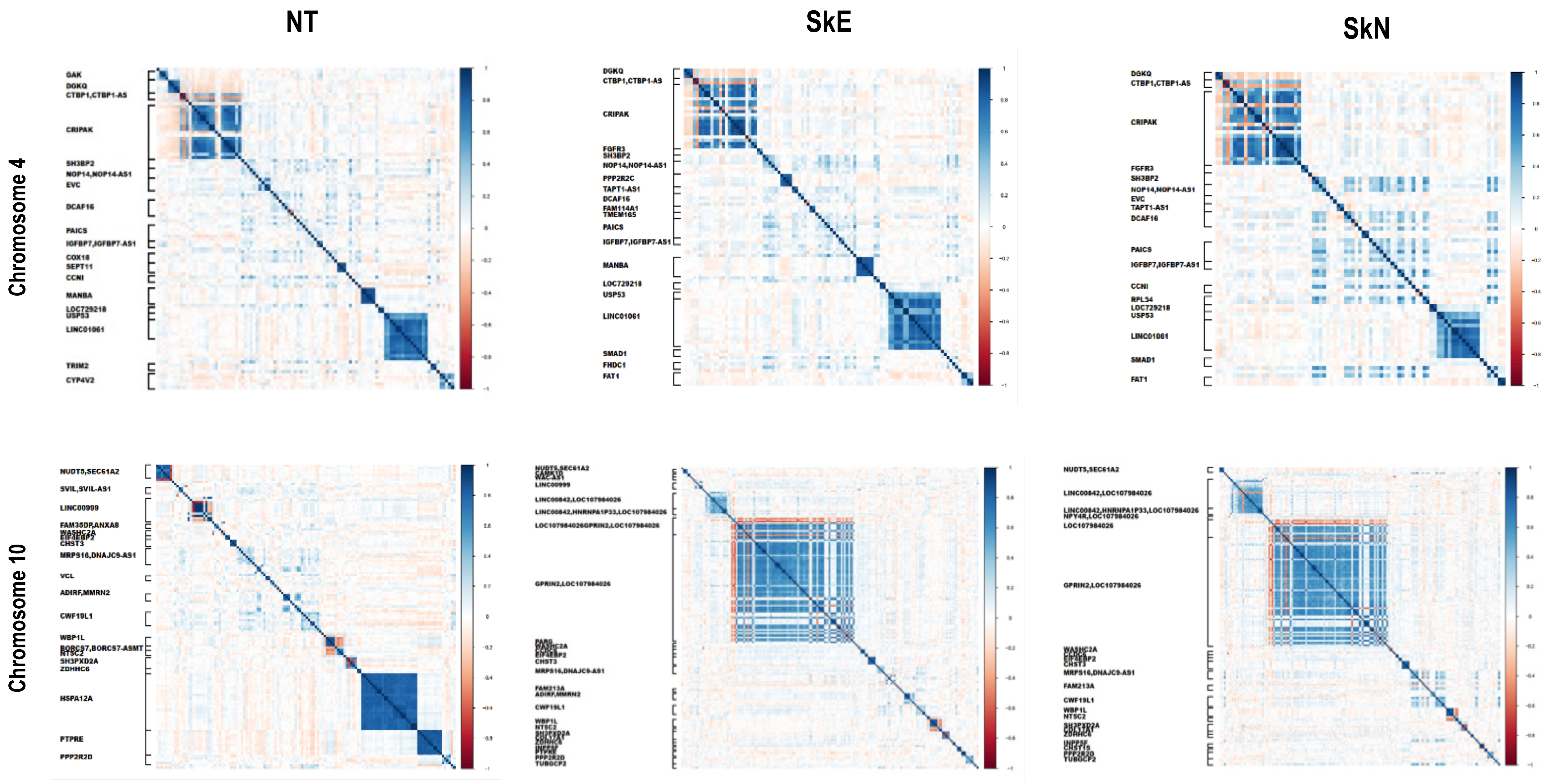


**S_Figure 8**. $VAF_{RNA}$ – $VAF_{RNA}$ correlations along genes with multiple significant SNVs within the same gene Strong positive (blue, in-phase), or negative (red, inverted phase) correlations are seen between the SNV positions residing in the same gene, indicating consistency in the $VAF_{RNA}$ estimation.
